# Recurrent Intestinal Obstruction Secondary to Crohn’s Disease in Dextrocardia With Situs Inversus Totalis: A Case Report

**DOI:** 10.7759/cureus.103517

**Published:** 2026-02-13

**Authors:** Muhammad Furqan Ubaid, Tamer Zahdeh, Akhila Sai Sree Cherukuri, Mahmoud El Hajj, Aiswarya Nair, Ahmed Irtaza, Michelle N Ritota, Hany H Abdallah, S. Elissa Altin

**Affiliations:** 1 Internal Medicine, Montefiore St. Luke’s Cornwall, Newburgh, USA; 2 Internal Medicine, Montefiore St. Luke's Cornwall, Newburgh, USA; 3 General Surgery, State University of New York (SUNY) Downstate Health, New York, USA; 4 Internal Medicine, University of Texas (UT) Health, San Antonio, USA; 5 Infectious Disease, University of Connecticut (UConn), Storrs, USA; 6 Internal Medicine, Hayatabad Medical Complex Peshawar, Peshawar, PAK; 7 Internal Medicine, St. George's University School of Medicine, True Blue, GRD; 8 Critical Care Medicine, Montefiore St. Luke's Cornwall, Newburgh, USA; 9 Internal Medicine, Yale New Haven Hospital, New Haven, USA

**Keywords:** congenital abnormalities, crohn’s disease (cd), dextrocardia, situs-inversus-totalis, small intestinal obstruction

## Abstract

Situs inversus totalis (SIT) is a rare congenital anomaly characterized by complete mirror-image transposition of the thoracic and abdominal organs. Although most individuals with SIT remain asymptomatic, acute abdominal pathology can pose significant diagnostic and surgical challenges due to reversed anatomical landmarks. Intestinal obstruction is an uncommon but serious complication in this population.

We report the case of a 46-year-old male with no prior knowledge of SIT who presented with a six-month history of intermittent abdominal pain, vomiting, and watery diarrhea, with recent worsening. Two months prior, he had been admitted for partial small bowel obstruction that was resolved with conservative management. Computed tomography (CT) during the current admission revealed recurrent small bowel obstruction with multiple dilated loops, distal ileitis, and mirror-image anatomy consistent with SIT. Initial conservative therapy again failed, prompting diagnostic laparoscopy with lysis of adhesions. The patient subsequently developed recurrent high-grade small bowel obstruction complicated by a pelvic abscess requiring exploratory laparotomy with resection of 25 cm of inflamed ileum and appendectomy. Histopathologic findings and chronicity of symptoms raised a strong suspicion for Crohn’s disease as the underlying etiology, and the patient was discharged with gastroenterology follow-up for further evaluation and management.

This case highlights the diagnostic complexity of acute abdominal conditions in patients with SIT and emphasizes the importance of early cross-sectional imaging for accurate diagnosis and operative planning. Awareness of reversed anatomy and consideration of underlying inflammatory etiologies are essential to avoid delays in management and optimize patient outcomes.

## Introduction

In humans, the thoracic and abdominal organs normally exhibit a consistent left-right (LR) orientation, a configuration known as situs solitus, which is established during embryogenesis through tightly regulated genetic and molecular mechanisms [1]. Disruptions in this process result in laterality disorders, which exist along a spectrum. At one extreme is situs inversus totalis (SIT), a rare congenital condition characterized by complete mirror-image transposition of both thoracic and abdominal viscera, while partial or indeterminate arrangements are classified as situs ambiguous [1,2].

Although most individuals with SIT remain asymptomatic, mirror-image anatomy poses significant diagnostic and therapeutic challenges, particularly in the setting of acute abdominal pathology [2,3]. Clinical localization of symptoms may be misleading, and physical examination alone is often insufficient, making cross-sectional imaging essential for accurate diagnosis and operative planning [1,3]. Surgical management is further complicated by reversed anatomic orientation, requiring modifications in operative approach and heightened spatial awareness, especially given the predominance of right-handed surgical techniques [1].

We report a case of situs inversus totalis complicated by recurrent small bowel obstruction secondary to suspected Crohn’s disease, resulting in repeated inflammatory episodes, adhesions, and fistula formation. This case highlights the diagnostic complexity and surgical considerations associated with acute abdominal conditions in patients with SIT and emphasizes the importance of early imaging and anatomical awareness to guide effective management.

## Case presentation

**Figure 1:**
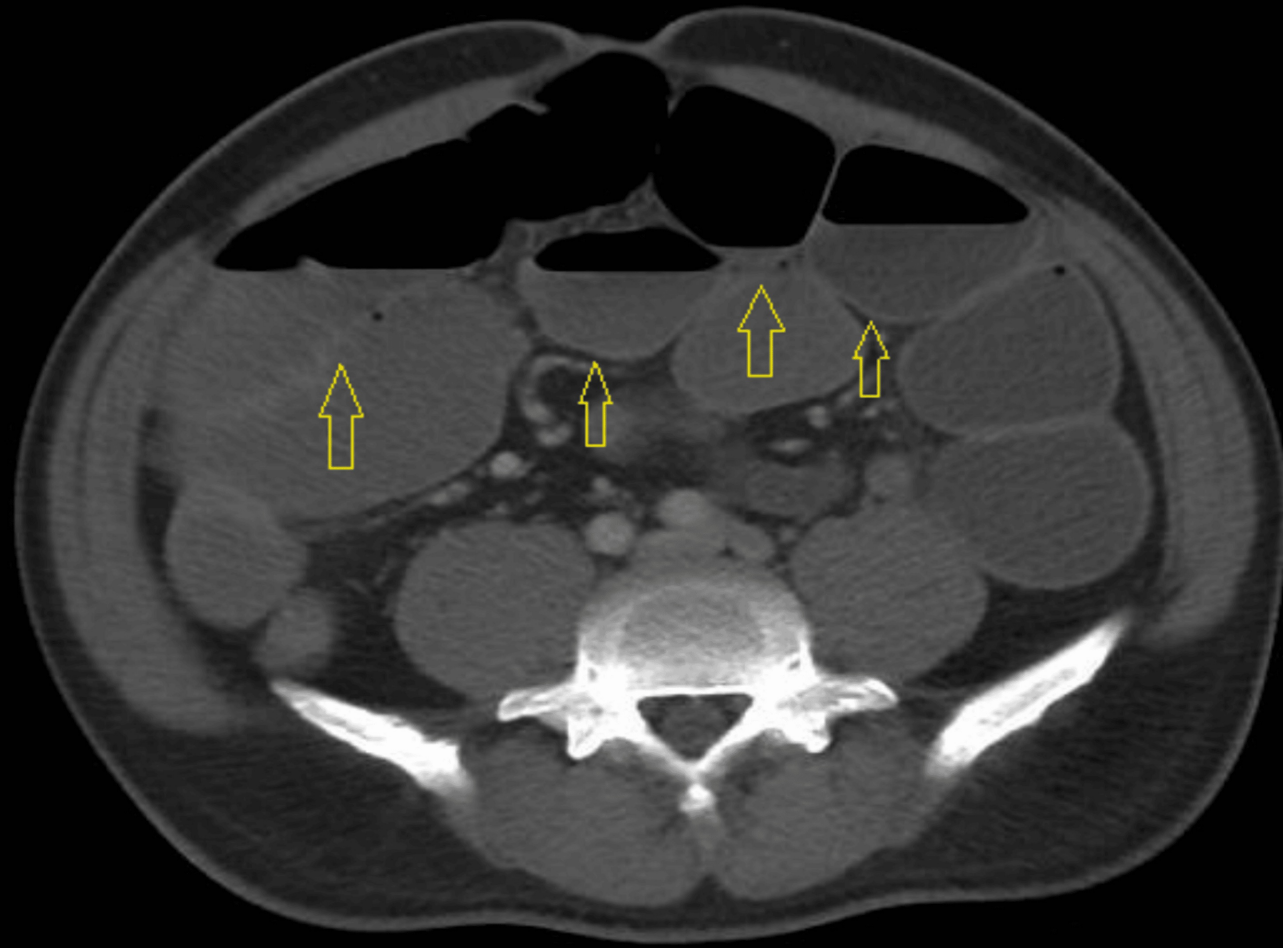
Axial contrast-enhanced CT scan of the abdomen demonstrating diffusely dilated, fluid-filled small-bowel loops with mild caliber change in the distal ileum (arrows), without a clear transition point. The presence of distal colonic gas suggests a partial distal small bowel obstruction versus ileus. No pneumatosis, portal venous gas, or free intraperitoneal air is identified.

**Figure 2:**
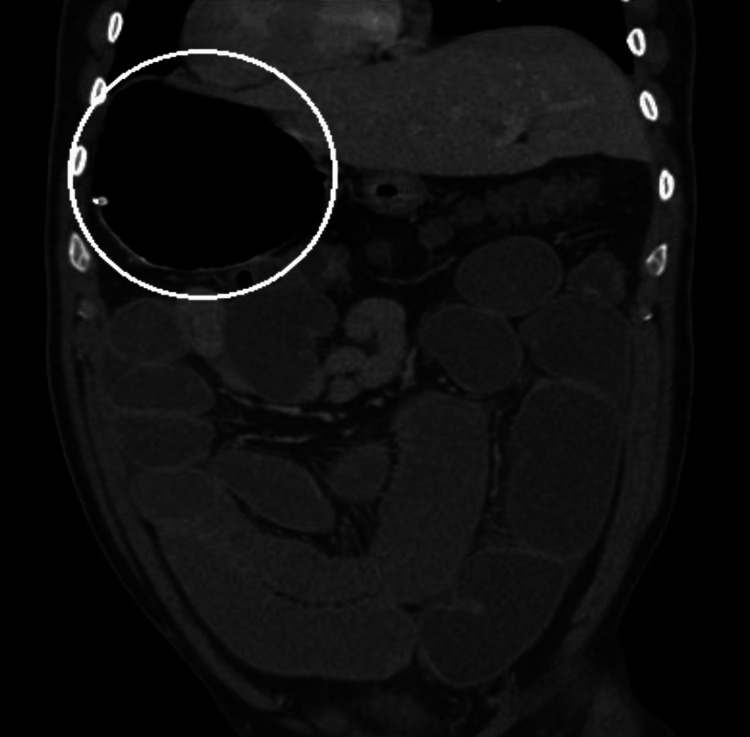
Coronal contrast-enhanced CT image demonstrating a right-sided distended stomach, consistent with mirror-image visceral orientation in situs inversus totalis, with associated dilated small-bowel loops in the mid-abdomen.

A 46-year-old male with a past medical history of hypertension presented with a six-month history of abdominal pain, intermittent vomiting, and watery diarrhea. He reported a two-month history of intermittent postprandial colicky abdominal pain occasionally accompanied by watery diarrhea. The last bowel movement was two days prior, and he denied passing flatus for 24 hours. He denied any surgical or family history of inflammatory bowel disease and had no extra-intestinal manifestations, such as joint pain or rash. At presentation, the patient was hemodynamically stable, afebrile, and normocytic. Physical examination revealed abdominal distension with mild diffuse tenderness to palpation, without guarding, rebound tenderness, or peritoneal signs. Mucous membranes were moist, and there was no clinical evidence of significant dehydration. Initial laboratory findings are summarized in Table 1. Computed tomography of the abdomen and pelvis revealed situs inversus totalis, fluid-filled and distended stomach, small bowel, and distal ileitis with residual mucosal edema (Figures 1, 2).

**Table 1:** Laboratory results on admission. AST: Aspartate Aminotransferase, ALT: Alanine Aminotransferase, INR: International Normalized Ratio

Laboratory Test	Patient Value	Reference Range	Interpretation
White Blood Cell Count	14.41 ×10³/µL	4.0–10.0 ×10³/µL	Elevated
Red Blood Cell Count	4.06 ×10⁶/µL	4.7–6.1 ×10⁶/µL	Decreased
Hemoglobin	11.0 g/dL	13.5–17.5 g/dL	Decreased
Hematocrit	35.7 %	41–53 %	Decreased
Mean Corpuscular Volume	87.9 fL	80–100 fL	Normal
Mean Corpuscular Hemoglobin	27.1 pg	27–33 pg	Normal
Mean Corpuscular Hemoglobin Concentration	30.8 g/dL	32–36 g/dL	Decreased
Red Cell Distribution Width	12.7 %	11.5–14.5 %	Normal
Platelet Count	388 ×10³/µL	150–400 ×10³/µL	Normal
Neutrophils (%)	79.9 %	40–75 %	Elevated
Absolute Neutrophil Count	11.52 ×10³/µL	1.8–7.7 ×10³/µL	Elevated
Lymphocytes (%)	14.8 %	20–40 %	Decreased
Absolute Lymphocyte Count	2.13 ×10³/µL	1.0–4.0 ×10³/µL	Normal
Monocytes (%)	4.6 %	5–12 %	Slightly decreased
Sodium	139 mmol/L	135–145 mmol/L	Normal
Potassium	4.8 mmol/L	3.5–5.1 mmol/L	Normal
Chloride	102 mmol/L	98–107 mmol/L	Normal
Carbon Dioxide	22 mmol/L	22–29 mmol/L	Normal
Blood Urea Nitrogen	11 mg/dL	7–20 mg/dL	Normal
Creatinine	1.02 mg/dL	0.7–1.3 mg/dL	Normal
Glucose	146 mg/dL	70–99 mg/dL	Elevated
Calcium	10.0 mg/dL	8.6–10.2 mg/dL	Normal
Magnesium	2.0 mg/dL	1.6–2.6 mg/dL	Normal
Phosphorus	3.3 mg/dL	2.5–4.5 mg/dL	Normal
Albumin	4.4 g/dL	3.5–5.0 g/dL	Normal
Total Protein	8.0 g/dL	6.0–8.3 g/dL	Normal
AST	23 U/L	10–40 U/L	Normal
ALT	22 U/L	7–56 U/L	Normal
Alkaline Phosphatase	58 U/L	44–147 U/L	Normal
Total Bilirubin	1.8 mg/dL	0.2–1.2 mg/dL	Elevated
Direct Bilirubin	0.34 mg/dL	<0.3 mg/dL	Slightly elevated
Indirect Bilirubin	1.5 mg/dL	0.2–0.8 mg/dL	Elevated
C-Reactive Protein	9.95 mg/dL	<0.5 mg/dL	Elevated
Erythrocyte Sedimentation Rate	28 mm/hr	0–20 mm/hr	Elevated
Prothrombin Time	12.9 sec	11–13.5 sec	Normal
INR	1.10	0.8–1.2	Normal

The initial differential diagnosis included acute appendicitis, ileus, and distal small-bowel obstruction. Given persistent obstructive symptoms despite bowel rest and intravenous fluids, imaging findings concerning for distal ileitis with possible inflammatory or adhesive obstruction, and the diagnostic uncertainty posed by situs inversus totalis anatomy, the decision was made to proceed with early diagnostic laparoscopy for both diagnostic clarification and potential therapeutic intervention. On hospital day three, a diagnostic laparoscopy was performed. Port placement was reversed to accommodate situs inversus totalis anatomy, with trocar positioning mirrored from standard orientation and the primary operating surgeon positioned contralaterally. Intraoperatively, dilated small-bowel loops were identified in the left lower quadrant, where a segment of distal ileum was adherent to the abdominal wall. The adhesions were lysed laparoscopically. The terminal ileum appeared inflamed but viable, without evidence of transmural necrosis, creeping fat, mesenteric thickening, serosal inflammation, ischemia, or free perforation. Following lysis of adhesions, bowel continuity and perfusion were preserved. Given the absence of necrosis or perforation and subsequent clinical improvement, non-resectional management was deemed appropriate at that time.

**Figure 3:**
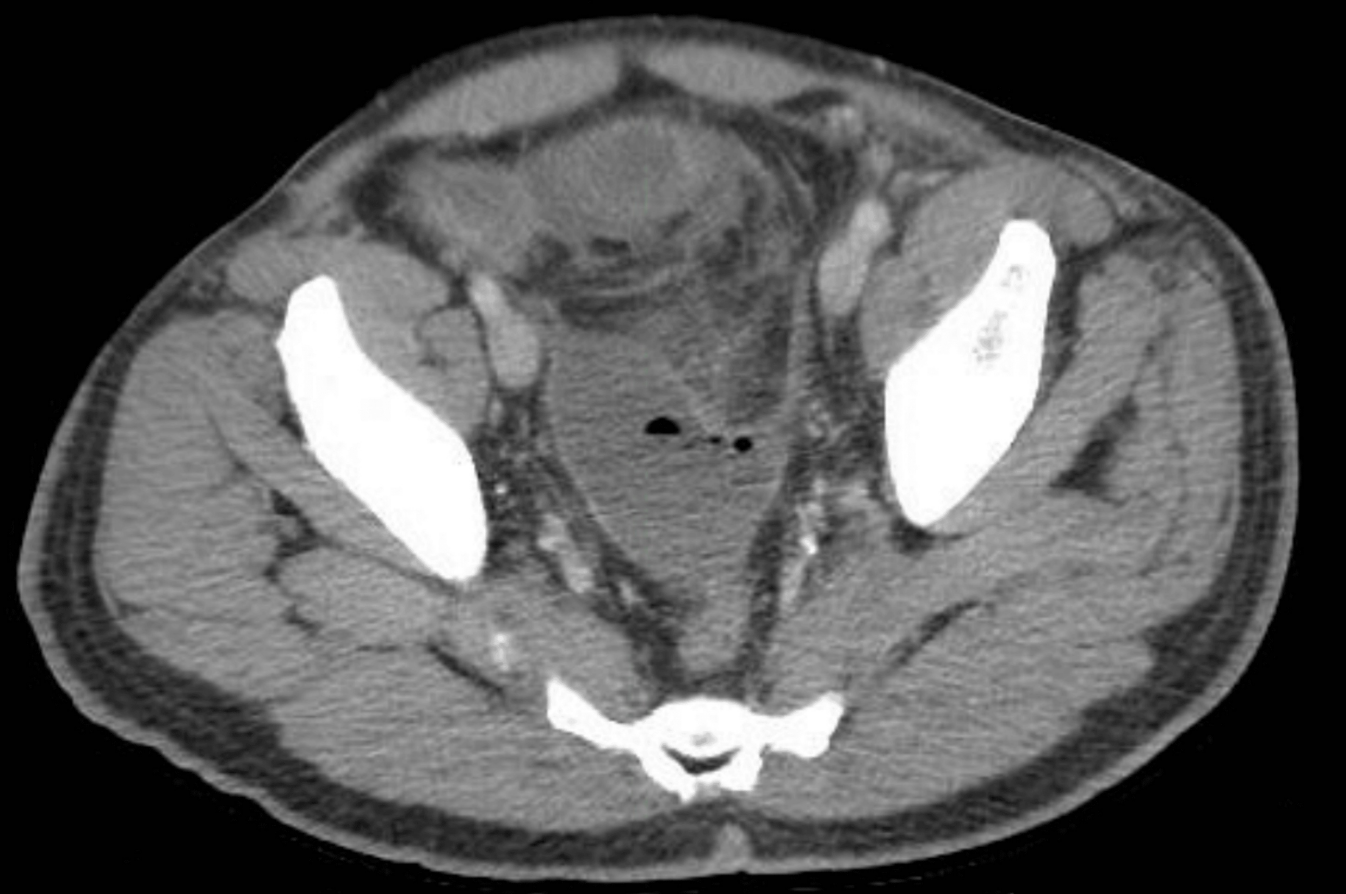
Axial contrast-enhanced CT image demonstrating a pelvic fluid collection with internal gas locules, measuring approximately 6.6 × 3.8 × 7.4 cm, findings concerning for a pelvic abscess in the setting of small bowel obstruction and inflammatory ileitis.

**Figure 4:**
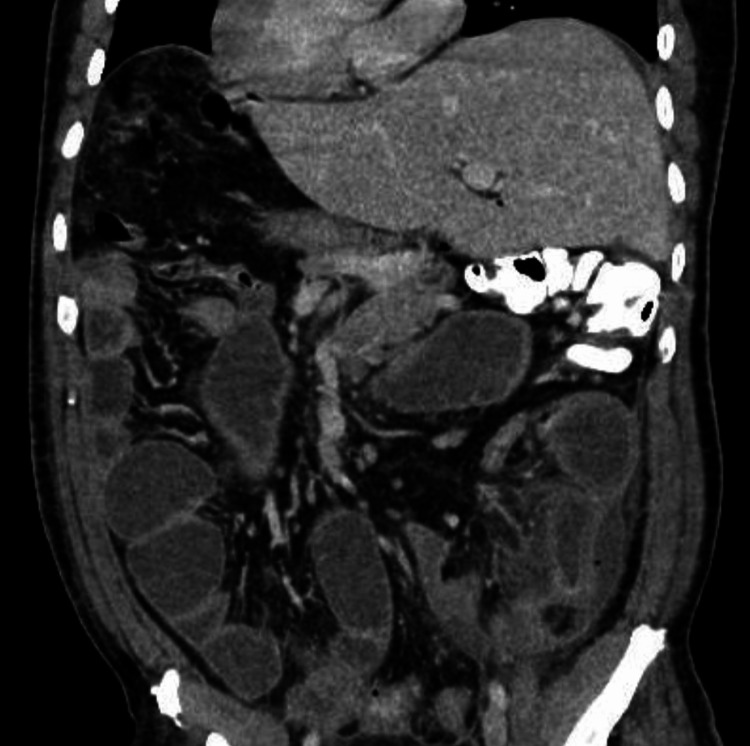
Coronal contrast-enhanced CT image demonstrating segmental small-bowel wall thickening in the left lower quadrant with marked adjacent mesenteric fat stranding, findings consistent with phlegmonous inflammatory change, suggestive of an underlying inflammatory etiology.

**Figure 5:**
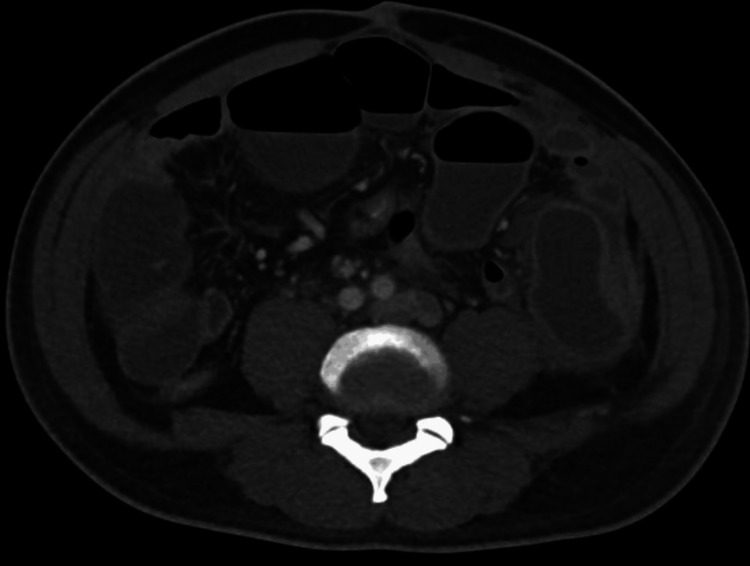
Axial contrast-enhanced CT image demonstrating marked dilation of proximal small-bowel loops with relative decompression of distal small bowel in the right lower quadrant and left paracolic gutter, findings consistent with a high-grade small bowel obstruction.

**Figure 6:**
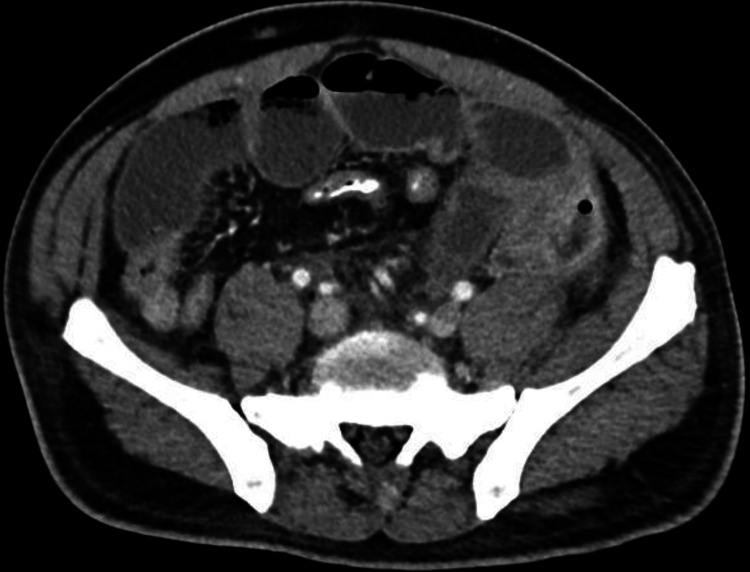
Axial contrast-enhanced CT image demonstrating an abnormal communication between adjacent small-bowel loops in the left paracolic gutter with associated mesenteric inflammatory changes and trace extraluminal fluid, findings concerning for an enteric fistula.

**Figure 7:**
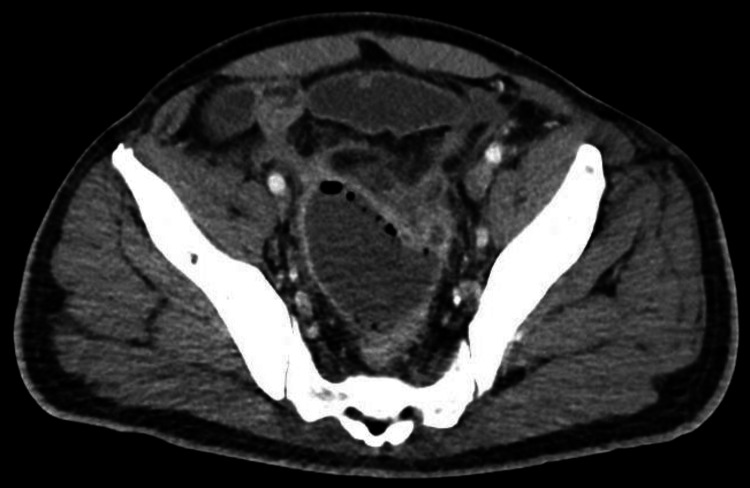
Axial contrast-enhanced CT image demonstrating interval enlargement of the central pelvic fluid collection, now measuring approximately 7.5 × 6.4 cm, with associated inflammatory changes, findings consistent with progression of a pelvic abscess.

On hospital day five, the patient developed worsening abdominal distension. A repeat CT of the abdomen and pelvis with contrast showed a pelvic fluid collection with small pockets of air, concerning for a developing abscess (Figure 3), as well as phlegmonous inflammation involving the small bowel in the left lower quadrant (Figure 4). Empiric intravenous piperacillin-tazobactam was initiated. A subsequent small bowel follow-through study revealed delayed contrast transit, raising concern for persistent partial small bowel obstruction versus ileus. Gastroenterology was consulted, and underlying ileitis was suspected, with Crohn’s disease considered as a possible etiology. Given the presence of acute high-grade small bowel obstruction and evolving intra-abdominal sepsis, endoscopic evaluation was deferred due to the risk of perforation. Infectious etiologies were considered. Stool studies, including *Clostridioides difficile* testing, were not obtained due to the absence of recent antibiotic exposure and lack of profuse inflammatory diarrhea at that time. Fecal calprotectin testing and cross-sectional enterography (CT or MR enterography) were planned for outpatient evaluation; however, these investigations were precluded by subsequent clinical deterioration requiring urgent surgical intervention. Subsequent imaging showed progression to high-grade small bowel obstruction (Figure 5), with a suspected fistulous tract in the left lower quadrant (Figure 6) and mild interval enlargement of the pelvic fluid collection (Figure 7).

CT-guided percutaneous drainage of the pelvic fluid collection was performed by interventional radiology, yielding 20 mL of foul-smelling purulent fluid. An 8.5-Fr pigtail catheter was placed and connected to gravity drainage, with an initial output of approximately 200 mL of purulent fluid in the first 24 hours and progressive decline thereafter. Cultures grew *Proteus mirabilis* and *Klebsiella oxytoca*, both susceptible to beta-lactam/beta-lactamase inhibitor therapy. The patient was continued on intravenous piperacillin-tazobactam with clinical improvement, including resolution of leukocytosis, decreased drain output, and improved oral tolerance. Given adequate source control, culture-directed susceptibility, and clinical stability, antibiotics were transitioned to oral amoxicillin-clavulanate (875/125 mg twice daily) to complete a 10-day total course, consistent with management of complicated intra-abdominal infection following source control.

**Figure 8:**
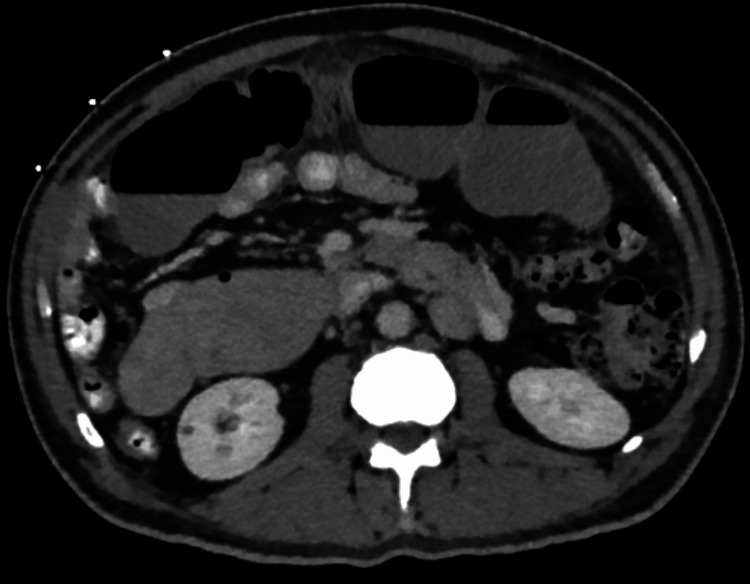
Axial contrast-enhanced CT image demonstrating markedly dilated proximal small-bowel loops with an abrupt caliber change, findings consistent with recurrent high-grade small bowel obstruction.

Two days after discharge, the patient presented with worsening abdominal distension, nausea, and vomiting. On examination, the abdomen was soft but distended with hypoactive bowel sounds. Laboratory tests in the ER revealed mild leukocytosis (12.4 K/µL). A CT scan of the abdomen and pelvis with contrast showed newly dilated loops of small bowel with a transition point in the mid-abdomen, consistent with recurrent high-grade small bowel obstruction (Figure 8).

**Figure 9:**
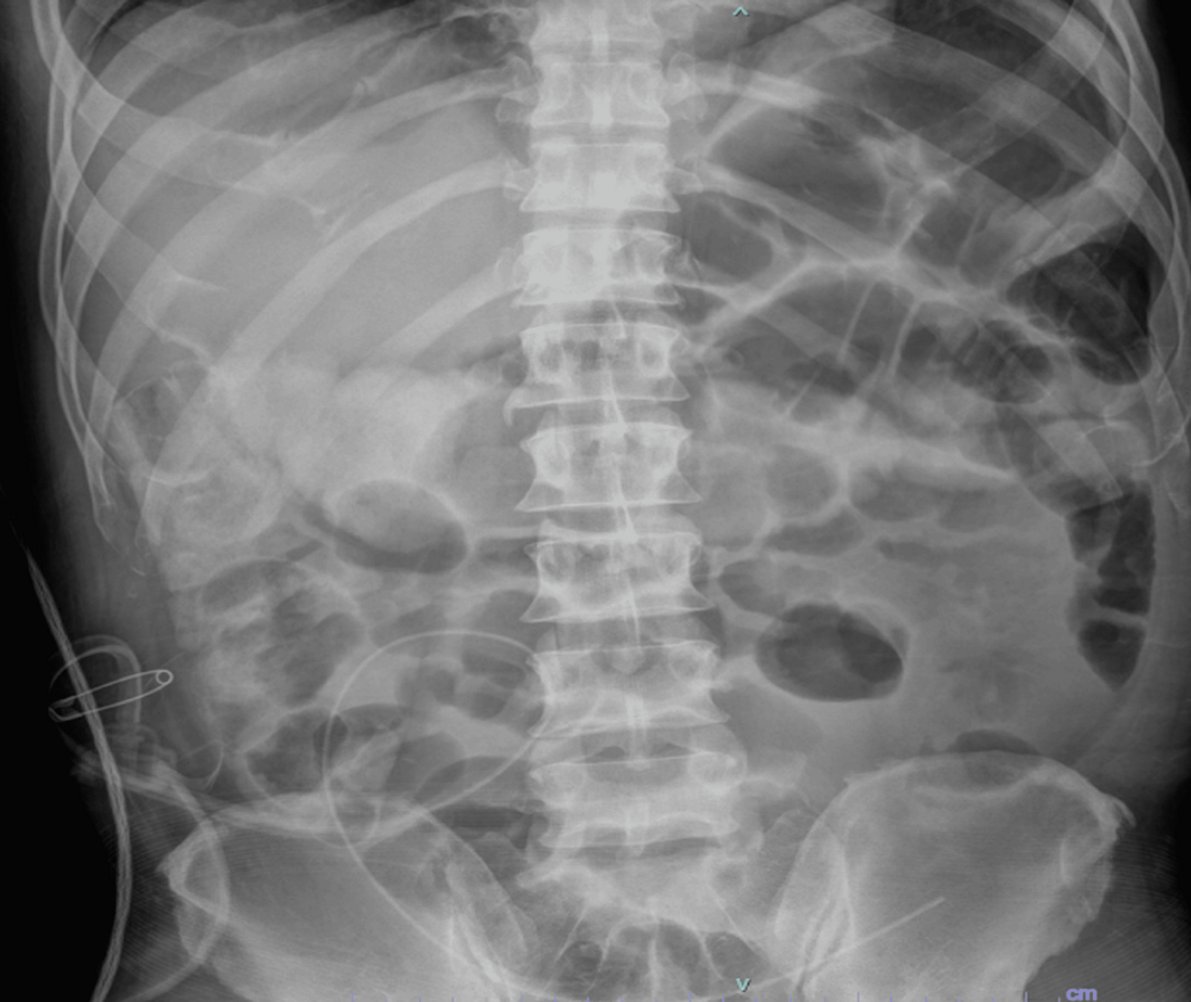
Abdominal radiograph showing persistently dilated small-bowel loops with residual contrast in the colon, suggestive of incomplete transit and ongoing small bowel obstruction.

Despite initial supportive management, the patient’s symptoms failed to improve. A subsequent kidney, ureter, and bladder (KUB) radiograph revealed dilated and air-filled loops of small bowel in the mid- to right abdomen with residual contrast in the colon, consistent with an ongoing small bowel obstructive pattern (Figure 9). Given the recurrent nature of his obstructions, the absence of a definitive diagnosis, and the presence of a clear transition point on imaging, the general surgery team elected to proceed with exploratory laparotomy for definitive diagnosis and management.

Intraoperatively, a transition point was identified in the left lower quadrant, associated with dense adhesions and inflammatory changes involving the small bowel. A segment of distal ileum, approximately 25 cm proximal to the ileocecal valve, was found adherent to the abdominal wall and contained a sealed microperforation. The pelvic fluid collection was therefore presumed to be secondary to a microperforation arising from the inflamed distal ileum, with subsequent localized contamination and abscess formation. Cross-sectional imaging did not demonstrate diffuse pneumoperitoneum or free intraperitoneal contrast extravasation, supporting a contained process. However, interval imaging raised suspicion for developing fistulous communication between the distal small bowel and the pelvic collection. An inflamed appendix was also noted on the left side, along with an enlarged mesenteric lymph node. There was no evidence of gross ischemia or diffuse intra-abdominal contamination beyond the walled-off perforated segment. An appendectomy was performed, followed by resection of approximately 25 cm of the involved ileum. A primary end-to-end ileoileal anastomosis was fashioned. Primary anastomosis was deemed appropriate given the absence of gross fecal contamination, diffuse peritonitis, or hemodynamic instability, with inflammation limited to a localized, walled-off microperforation and preservation of healthy bowel margins with adequate perfusion.

Histopathologic examination of the resected ileum revealed crypt abscesses, pyloric metaplasia, blunted villi, and infiltration of neutrophils and lymphocytes within the lamina propria. The previously placed transgluteal pelvic drain from the prior hospitalization was removed. The abdominal fascia was closed, while the skin was left open due to contamination risk and managed with a negative-pressure wound therapy system. A left paracolic drain was placed for continued postoperative drainage. Postoperatively, the patient was treated with intravenous cefazolin and metronidazole, along with patient-controlled analgesia for pain management. Delayed primary skin closure was planned. His postoperative course focused on monitoring for return of bowel function, resolution of infection, and outpatient gastroenterology follow-up for further evaluation and management of suspected Crohn’s disease.

## Discussion

Etiology and clinical implications of situs inversus totalis

Situs inversus totalis (SIT) is a rare congenital laterality disorder characterized by complete mirror-image transposition of thoracic and abdominal organs. Embryologically, SIT arises from disruption of normal left-right axis determination during early development, rather than from abnormalities of midgut rotation alone. Proper laterality is established through leftward nodal flow generated by motile cilia within the left-right organizer (LRO) at the primitive node, which drives asymmetric activation of signaling pathways including NODAL, LEFTY, and PITX2. Failure of this process results in a global reversal of visceral organ positioning. Abnormal intestinal rotation, including mirrored or atypical midgut rotation around the superior mesenteric artery, is therefore a downstream consequence of altered left-right patterning rather than the primary embryologic defect [4,5].

Although SIT is often clinically silent, its anatomic configuration has important implications when acute abdominal pathology occurs. Reversed organ positioning can predispose patients to diagnostic delays and surgical challenges. In particular, clockwise midgut rotation in SIT has been associated with an increased risk of volvulus and other obstructive processes [6,7]. More commonly, SIT complicates clinical localization of symptoms, as pain may present contralateral to expected anatomic landmarks, increasing the risk of misdiagnosis or delayed intervention [1].

Diagnostic challenges in acute abdominal pathology

Most individuals with SIT remain unaware of their condition until it is incidentally identified during imaging or surgery [1,2]. When acute abdominal symptoms develop, clinicians may be misled by atypical pain localization. While the symptoms of small bowel obstruction, like nausea, vomiting, abdominal pain, and distension, are similar in patients with situs solitus and SIT, the expected physical findings may be reversed, complicating bedside assessment.

In our case, abdominal CT played a pivotal role by simultaneously identifying small bowel obstruction and confirming complete visceral transposition. Cross-sectional imaging is essential in such patients, as it allows accurate localization of pathology and guides operative planning [1-3]. Additional clues to SIT, such as dextrocardia on chest radiography or a right-sided gastric bubble, may further prompt recognition of abnormal anatomy. In diagnostically ambiguous cases, diagnostic laparoscopy may provide definitive confirmation of organ orientation and underlying pathology [3].

Prior case reports have highlighted similar diagnostic pitfalls in patients with situs inversus totalis (SIT). Abdulla et al. described a 65-year-old male with SIT presenting with left lower quadrant pain who was found to have acute appendicitis on CT scan [3]. Additional reports have described left-sided appendicitis and appendicular peritonitis in patients with SIT, as well as small bowel obstruction caused by internal hernias or volvulus, emphasizing how mirrored anatomy can delay diagnosis and definitive management [8-10]. Collectively, these cases emphasize that early imaging and heightened anatomical vigilance are critical to avoid diagnostic error.

Crohn’s disease as a contributing etiology

Crohn’s disease (CD) is a chronic inflammatory bowel disease characterized by transmural inflammation and a predilection for the terminal ileum [11,12]. Common clinical features include abdominal pain and diarrhea, and intestinal obstruction is a frequent complication resulting from recurrent inflammation, fibrosis, stricture formation, fistula development, or abscess formation [12-14]. Appendiceal involvement has been described in Crohn’s disease and is often associated with more extensive intestinal inflammation [15].

In our patient, distal ileitis was identified on initial imaging, prompting gastroenterology consultation and consideration of CD. Operative findings, including inflammatory adhesions, a sealed perforation, phlegmon, abscess formation, and an inflamed appendix, further supported an inflammatory etiology. Histopathologic examination of the resected ileal segment demonstrated crypt abscesses, pyloric metaplasia, blunted villi, and chronic inflammatory infiltrates within the lamina propria. Transmural inflammation, fissuring ulcers, and non-caseating granulomas were not identified, and there was no established fibrotic stricture, consistent with early or evolving inflammatory bowel disease rather than long-standing Crohn’s disease. These histologic features have been shown to occur more frequently in Crohn’s disease and suspected Crohn’s disease compared with non-inflammatory controls [16]. Histologic evaluation of the appendix revealed acute and chronic inflammatory changes without granulomas or transmural involvement, favoring reactive appendiceal inflammation in the setting of adjacent ileal disease rather than primary acute appendicitis.

Although non-caseating granulomas are considered specific for Crohn’s disease, they are absent in many cases, and diagnosis relies on a constellation of clinical, radiologic, and histopathologic findings rather than a single defining feature [16,17]. Taken together, the clinical presentation, imaging abnormalities, operative findings, and histology in this case were most consistent with suspected Crohn’s disease as the underlying driver of recurrent obstruction, while appropriately warranting longitudinal gastroenterology follow-up rather than definitive diagnosis based on pathology alone.

Surgical considerations in SIT

Surgical management of intestinal obstruction in patients with situs inversus totalis requires careful preoperative planning and intraoperative adaptability. Contrast-enhanced CT is critical for defining anatomic orientation and identifying the site and cause of obstruction [1]. During laparoscopy, port placement and surgeon positioning often must be reversed, and operating from the contralateral side may be necessary to maintain ergonomic efficiency [2,3].

Open surgery remains appropriate in complex cases involving dense adhesions, perforation, or extensive inflammation. Surgeons must anticipate reversed vascular and visceral relationships and adjust standard operative strategies accordingly. Multidisciplinary coordination among surgeons, radiologists, and anesthesiologists familiar with SIT anatomy is essential to reduce operative risk and optimize outcomes [2].

## Conclusions

Situs inversus totalis is a rare congenital condition that can significantly complicate the diagnosis and management of acute abdominal pathology. This case illustrates how recurrent small bowel obstruction in the setting of mirror-image anatomy may obscure clinical localization and delay definitive treatment without early cross-sectional imaging. It also highlights the importance of considering underlying inflammatory bowel disease as a potential contributing etiology in patients with recurrent obstruction, even in anatomically atypical presentations. Heightened clinical awareness and multidisciplinary coordination are essential to optimize outcomes in this uncommon but challenging clinical scenario.
